# Technical note: Extended longitudinal and lateral 3D imaging with a continuous dual-isocenter CBCT scan

**DOI:** 10.1002/mp.16234

**Published:** 2023-02-01

**Authors:** Tess Reynolds, Sepideh Hatamikia, Yiqun Q. Ma, Owen Dillon, Grace Gang, J. Webster Stayman, Ricky O’Brien

**Affiliations:** 1 Faculty of Medicine and Health, University of Sydney, Sydney, Australia; 2 Austrian Center for Medical Innovation and Technology, Wiener Neustadt, Austria; 3 Research Center for Medical Image Analysis and Artificial Intelligence (MIAAI), Department of Medicine, Danube Private University, Krems, Austria; 4 Johns Hopkins University, Baltimore, USA; 5 Department of Radiology, University of Pennsylvania, Philadelphia, USA; 6 School of Health and Biomedical Sciences, RMIT University, Melbourne, Australia

**Keywords:** cone beam CT, image reconstruction, orthopaedics

## Abstract

**Background:**

The clinical benefits of intraoperative cone beam CT (CBCT) during orthopedic procedures include (1) improved accuracy for procedures involving the placement of hardware and (2) providing immediate surgical verification.

**Purpose:**

Orthopedic interventions often involve long and wide anatomical sites (e.g., lower extremities). Therefore, in order to ensure that the clinical benefits are available to all orthopedic procedures, we investigate the feasibility of a novel imaging trajectory to simultaneously expand the CBCT field-of-view longitudinally and laterally.

**Methods:**

A continuous dual-isocenter imaging trajectory was implemented on a clinical robotic CBCT system using additional real-time control hardware. The trajectory consisted of 200° circular arcs separated by alternating lateral and longitudinal table translations. Due to hardware constraints, the direction of rotation (clockwise/anticlockwise) and lateral table translation (left/right) was reversed every 400°. X-ray projections were continuously acquired at 15 frames/s throughout all movements. A whole-body phantom was used to verify the trajectory. As comparator, a series of conventional large volume acquisitions were stitched together. Image quality was quantified using Root Mean Square Deviation (RMSD), Mean Absolute Percentage Deviation (MAPD), Structural Similarity Index Metric (SSIM) and Contrast-to-Noise Ratio (CNR).

**Results:**

The imaging volume produced by the continuous dual-isocenter trajectory had dimensions of L = 95 cm × W = 45 cm × H = 45 cm. This enabled the hips to the feet of the whole-body phantom to be captured in approximately half the imaging dose and acquisition time of the 11 stitched conventional acquisitions required to match the longitudinal and lateral imaging dimensions. Compared to the stitched conventional images, the continuous dual-isocenter acquisition had RMSD of 4.84, MAPD of 6.58% and SSIM of 0.99. The CNR of the continuous dual-isocenter and stitched conventional acquisitions were 1.998 and 1.999, respectively.

**Conclusion:**

Extended longitudinal and lateral intraoperative volumetric imaging is feasible on clinical robotic CBCT systems.

## 1 | INTRODUCTION

The clinical benefits of performing cone beam CT (CBCT) imaging during orthopedic interventions include, (1) improved accuracy for procedures involving the placement of hardware^1,2^ and (2), providing immediate surgical verification of the procedure in-room,^3,4^ thereby reducing the probability of a secondary corrective surgery when an issue is identified on post-operative imaging. One of the barriers for the continued expansion of intraoperative CBCT imaging during orthopedic interventions, however, is the limited CBCT field-of-view.

During orthopedic interventions, for example, the current CBCT field-of -view prevents anatomical sites that are both long and wide from being captured in a single volumetric image intraoperatively. This prevents 3D-3D registration between the anatomy in the operating room with the anatomy in the planning images acquired prior to the procedure, as well as intraoperative assessment before the procedure is completed. To address these issues, efforts have been directed towards expanding the CBCT field-of -view.^5^ However, most work completed to date on robotic CBCT systems has been focused on single direction expansion (i.e., only lateral or longitudinal expansion). For example, lateral CBCT expansion on robotic CBCT systems has been previously demonstrated using offset^6^ and rotated detector^7^ configurations. Similarly, longitudinal CBCT expansion on robotic CBCT systems has been achieved using non-circular trajectories such as the multi-turn reverse helical^8,9^ and line-ellipse-line trajectory.^10^ As such, there remains an unmet need to develop volumetric imaging techniques that enable simultaneous longitudinal and lateral expansion of the field-of -view on robotic CBCT imaging systems.

Here, we look to combine offsetting the detector via lateral table translations with a modified line-ellipseline trajectory to form a continuous dual-isocenter imaging trajectory that provides both longitudinal and lateral CBCT field-of -view expansion. To demonstrate the potential of this technique to extend the intraoperative CBCT field-of -view, we implement the continuous dual-isocenter imaging trajectory on a clinical robotic CBCT system, using additional real-time control, and acquire images from the hips to the feet of a whole-body phantom.

## 2 | MATERIALS AND METHODS

The Siemens ARTIS pheno floor-mounted robotic CBCT imaging system (Siemens Healthcare GmbH, Erlangen, Germany) was used to image from the hip to feet of a whole-body phantom (Kyoto Kagaku Co., Ltd.) with a continuous dual-isocenter. The continuous dual-isocenter acquisition was achieved with additional real-time control hardware provided by Siemens, known as the Siemens Test Automation Control System (TACS). A series of conventional acquisitions covering the same anatomical volume were acquired and stitched together, acting as comparator for the continuous dual-isocenter acquisition.

### 2.1 | Continuous dual-isocenter CBCT acquisition

**FIGURE 1:**
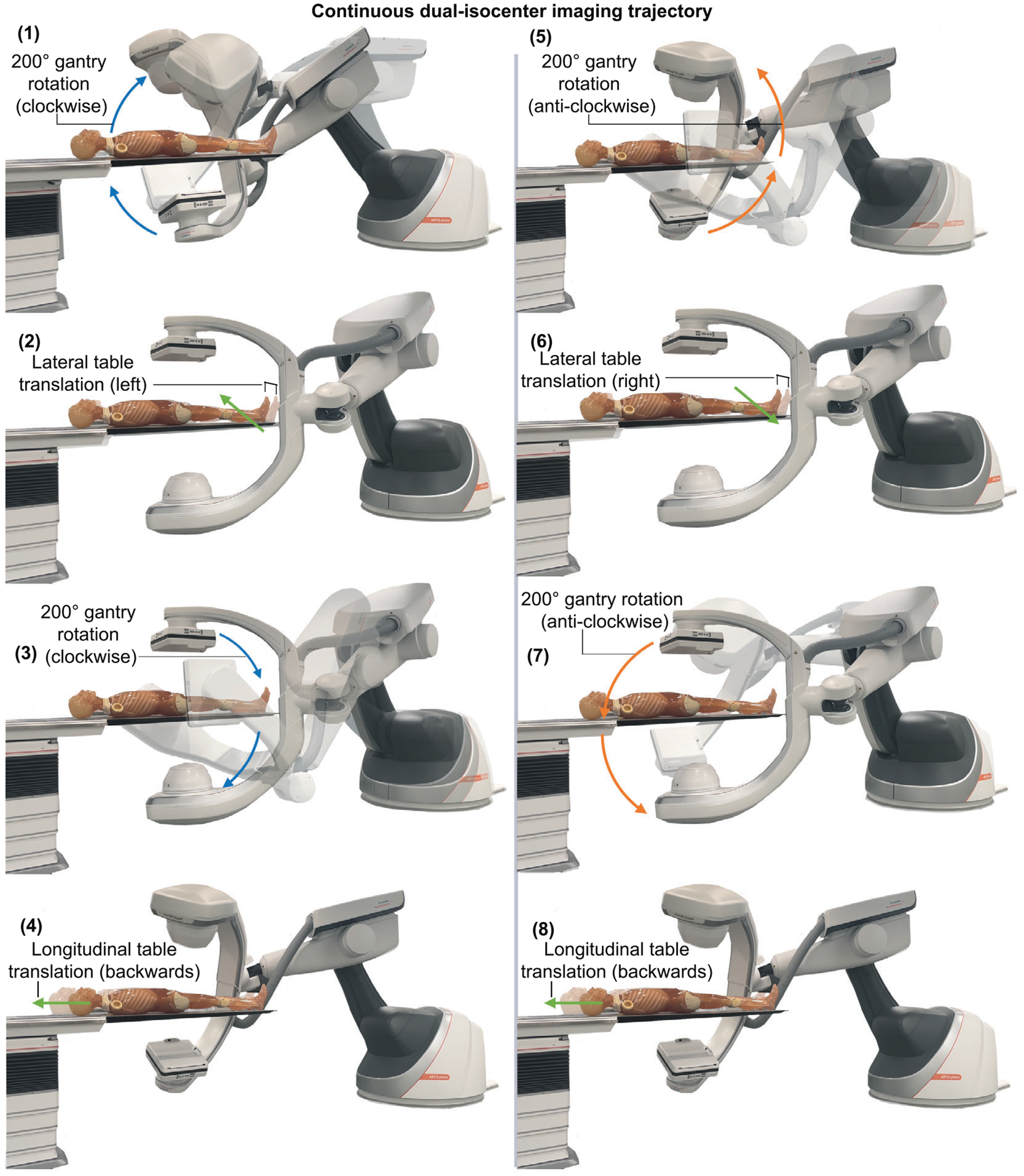
The individual gantry rotation and table translations components, labelled (1)–(8), that combine to form the continuous dual-isocenter 3D CBCT imaging trajectory for extending the lateral and longitudinal intraoperative 3D coverage with a fixed-room robotic imager.

The continuous dual-isocenter acquisition consists of 200° circular arcs completed by the C-arm gantry separated by alternating lateral (160 mm) and longitudinal (130 mm) table translations, Figure 1. The lengths of the table translations were selected to assist in minimizing the “overscan” regions while ensuring complete coverage of the imaging target in the presence of potential inaccuracies of the table or gantry positioning. Due to hardware constraints of the C-arm gantry, the direction of rotation (clockwise/anticlockwise) and lateral table translation (left/right) was reversed every 400°. The longitudinal table translation was always backwards, away from the C-arm gantry. Movements of the C-arm gantry and table were programmed and controlled through the TACS using software commands sent via a C# Dynamic Link Library. To demonstrate the maximum possible longitudinal coverage with the continuous dual-isocenter acquisition, the C-arm gantry was initially positioned at an angle of 40° to the table, with the table positioned as close as possible to the C-arm gantry without instigating a system collision warning or causing a collision with the phantom. In this configuration, the table movements were able to be repeated seven times over the course of the acquisition, resulting in a total longitudinal table translation of 91 cm. The longitudinal coverage of the final volume is determined by the total number of individual longitudinal table translations completed and the detector length of the system.

In clinical settings, the C-arm gantry of robotic CBCT imaging systems can rotate with a velocity of up to 45°/s. However, when operating the robotic CBCT imaging system via the TACS, the gantry rotation velocity is restricted to a maximum of 20°/s. As a result, the continuous dual-isocenter acquisition in its current form has a scan time of 5 min, not including pre-imaging steps such as selecting the isocenter and completing collision avoidance checks. It is predicted that these pre-imaging steps would add approximately 2 min to the total scan time if automated in a similar fashion to current conventional volumetric acquisition protocols. X-ray projections were acquired continuously throughout all C-arm gantry rotations and table translations at 90 kV, 0.5 mAs, and 15 frames per second, resulting in a total of approximately 3000 projections.

To enable assessment of the reproducibility of the continuous dual isocenter imaging trajectory (table and C-arm movements), the system properties (position, velocity, and acceleration) of the table and C-arm were recorded during four consecutive continuous dualisocenter acquisitions.

### 2.2 | Conventional large volume CBCT acquisition

The comparator method was an in-built lateral extension protocol available on the Siemens ARTIS pheno named 10s Dyna CT Large Volume (Siemens Healthineers, GmbH), referred to throughout as simply the conventional acquisition. The conventional acquisition provides an imaging volume that has 17.5 cm of longitudinal coverage and 45 cm of lateral coverage, from 546 projections acquired at 90 kV and 0.5 mAs. A series of 11 conventional acquisitions were sequentially acquired, separated by manual longitudinal table translations, to capture the entire lower extremities of a whole-body phantom, resulting in a total of 6006 projections being acquired. The conventional acquisition has a “beam on” acquisition time of 10 s. However, there is an additional 2 min of automated set-up prior to the acquisition commencing that includes selecting the isocenter and completing collision avoidance checks. If there is a table translation between acquisitions, as was the case in this study, the additional automated set up checks are enforced by the system and repeated every acquisition, significantly increasing overall acquisition time. The conventional acquisitions were reconstructed using the in-built algorithm provided on the Siemens ARTIS pheno. Currently, stitching more than two reconstructed volumes together on the Siemens ARTIS pheno is not possible. As such, the 11 individual conventional reconstruction volumes were manually aligned and stitched together offline using MATLAB (MathWorks, USA) to form the final volume used for comparison.

### 2.3 | Geometry calibration and image reconstruction of the continuous dual-isocenter acquisition

To facilitate the continuous dual-isocenter trajectory and account for inaccuracies in the recorded geometry from the system, accurate geometric calibration of each project was provided via a 3D-2D registration.^11^ Here, the stitched conventional volume acted as the 3D registration target. For each projection in the continuous dual-isocenter acquisition, the Covariance Matrix Adaption Evolution Strategy (CMA-ES) algorithm was used to identify the projection matrix that best matched the projected registration target with the measured projection. The gradient correlation was used as the similarity metric.

For image reconstruction, a model-based iterative reconstruction algorithm was adopted to best accommodate the non-circular geometrics, using a Penalized Weighted Least Squares (PWLS) objective with quadratic penalty^12^ and ordered subsets.^13^ 90 iterations were run with 14 subsets and 3066 total projections. The forward model of the mean photon measurements after attenuation is formulated conventionally under the assumption of monoenergetic x-ray beams:

(1) $$\overset{‾}{y}=D\{g\}\;exp(-I),\;I=A\mu$$

where $\overset{‾}{y}$ denotes the mean measurements, D{⋅} denotes an operator that places a vector along the diagonal of a matrix, *g* denotes measurement-dependent gains, *l* denotes line integrals, **A** denotes the linear projection operator, and denotes the linear attenuation coefficients of the object. We adopt a Poisson model for the variations between noisy and mean measurements.

The PWLS objection function is formulated as:

(2) $$\overset{ˆ}{\mu }=\underset{\mu \geq 0}{argmin}\frac{1}{2}A\mu -I_{W}^{2}+R(\mu ),\;R(\mu )=\beta_{R}\Psi_{R}\mu_{p_{R}}^{p_{R}}$$

Where $\overset{ˆ}{\mu }$ denotes the estimate of the linear attenuation coefficients, **W** denotes a diagonal weighting matrix representing the fidelity of the measurements, and R(μ) denotes the regularization term that penalizes large differences in a first-order neighbourhood around each voxel to encourage smoothness in the image. The penalty includes a scalar regularization strength term $\beta_{R}$ and a general *p*-norm operator $\Psi_{R}$ In this case, 2-norm is used (*p_R_* = 2) resulting in a quadratic penalty. Finally, the measurement fidelity **W** is approximated as:

(3) $$W_{i}=\left\{\begin{array}{c} 0,y_{i}\;\mathrm{is saturated}\\ y_{i},\;\mathrm{otherwise} \end{array}\right.$$

where *y* denotes the noisy measurements, and *i* indexes the diagonal elements in **W** and individual measurements in *y*. Effectively, non-saturated and less attenuated measurements are assigned higher fidelity values.

The projections in each subset are uniformly sampled throughout the entire scan. Image reconstruction for the continuous dual-isocenter acquisition took 3 h to complete.

### 2.4 | Reconstructed image quality and geometric accuracy

The image quality of the reconstructed volumes from both the continuous dual-isocenter and stitched conventional acquisitions was quantified using four image quality metrics. Specifically, Root Mean Square Deviation (RMSD):

(4) $$RMSD=\sqrt{\frac{1}{N}\sum_{j=1}^{N}{\left(x_{\mathrm{reference}}(j)-x_{\mathrm{comparison}}(j)\right)}^{2}}$$

where *N* is the number of voxels in the region of interest (ROI), *x_reference_*(*j*) is the j’th voxel in the reference volume ROI, *x_comparison_*(*j*) is the j’th voxel in the comparison volume ROI. Note that we compute the CBCT images and in turn the RMSD in units of cm^−1^.

Mean Absolute Percentage (MAPD):

(5) $$MAPD=\frac{100}{n}\sum_{j=1}^{n}\left|\frac{x_{\mathrm{reference}}(j)-x_{\mathrm{comparison}}(j)}{x_{\mathrm{reference}}(j)}\right|$$

where *n* < *N* is the number of non-air voxels in the ROI (classified as voxels with attenuation below 0.01*cm*^−1^).

Structural Similarity Index Metric (SSIM):

(6) $$\mathrm{SSIM}=\frac{1}{M}\sum_{M}\frac{\left(2\mu_{\mathrm{reference}}\mu_{\mathrm{comparison}}+c_{1}\right)\;\left(2\sigma_{\mathrm{reference,comparison}}+c_{2}\right)}{\left(\mu_{\mathrm{reference}}^{2}+\mu_{\mathrm{comparison}}^{2}+c_{1}\right)\;\left(\sigma_{\mathrm{reference}}^{2}+\sigma_{\mathrm{comparison}}^{2}+c_{2}\right)}$$

**FIGURE 2:**
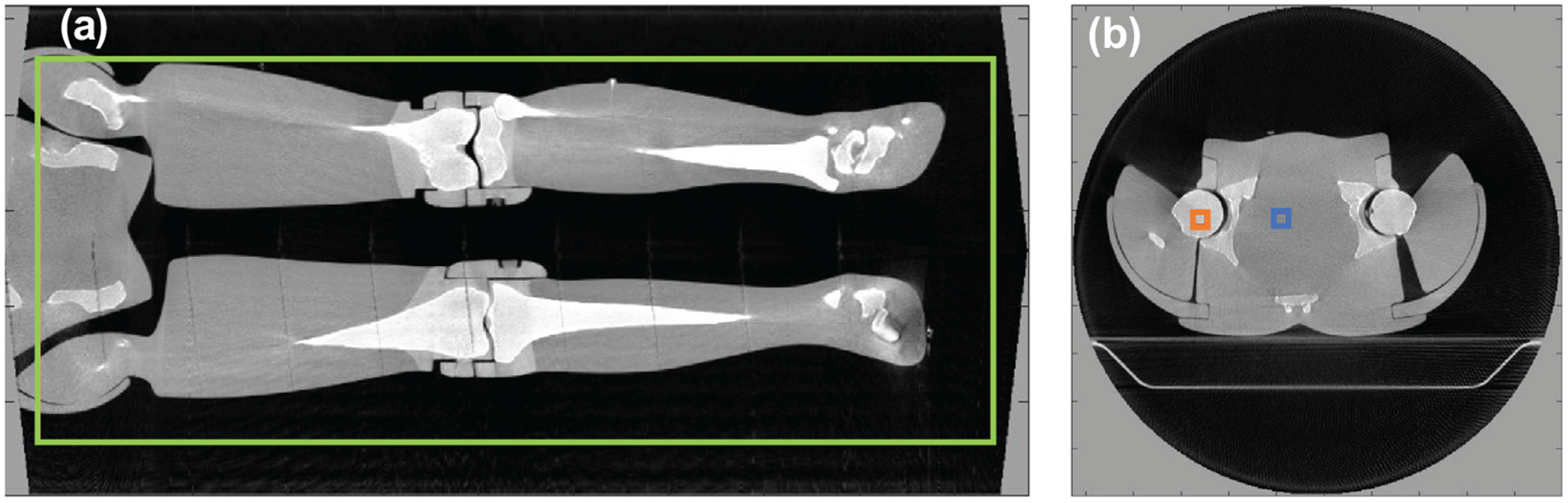
(a) The region of interest (green) used to compute the root mean square deviation, mean absolute percentage, and structural similarity index metric. (b) The foreground (orange) and background (blue) regions of interest used to compute the contrate to noise ratio.

Is computed over M windows inside the region $\mu_{\mathrm{reference}}$ and $\mu_{\mathrm{comparison}}$ are voxel value means in that window, $\sigma_{\mathrm{reference}},\sigma_{\mathrm{comparison}}$ and $\sigma_{\mathrm{reference,comparison}}$ are standard deviation and joint deviation, *c*_1_ = (0.1*L*)^2^ and *c*_2_ = (0.3*L*)^2^ are weighting factors with *L* the dynamic range in this case 1 as the volumes were normalised prior to computing SSIM. Structural similarity is unitless and varies from 0 to 1 with 1 being identical images. The ROI used for calculating the RMSD, MAPD and SSIM is shown in Figure 2a.

Contrast-to-Noise Ratio (CNR):

(7) $$CNR=\frac{\left|\mu_{fg}-\mu_{bg}\right|}{\sigma }$$

where $\mu_{fg}$ and $\mu_{bg}$ were the mean voxel values of the foreground and background 10 × 10 × 10 mm^3^ regions of interest, respectively, and σ was the standard deviation within the background region of interest. Note that CNR is dimensionless but can be understood as the number of standard deviations separating the foreground and background mean values. The ROI used for calculating the CNR is shown in Figure 2b.

## 3 | RESULTS

**FIGURE 3:**
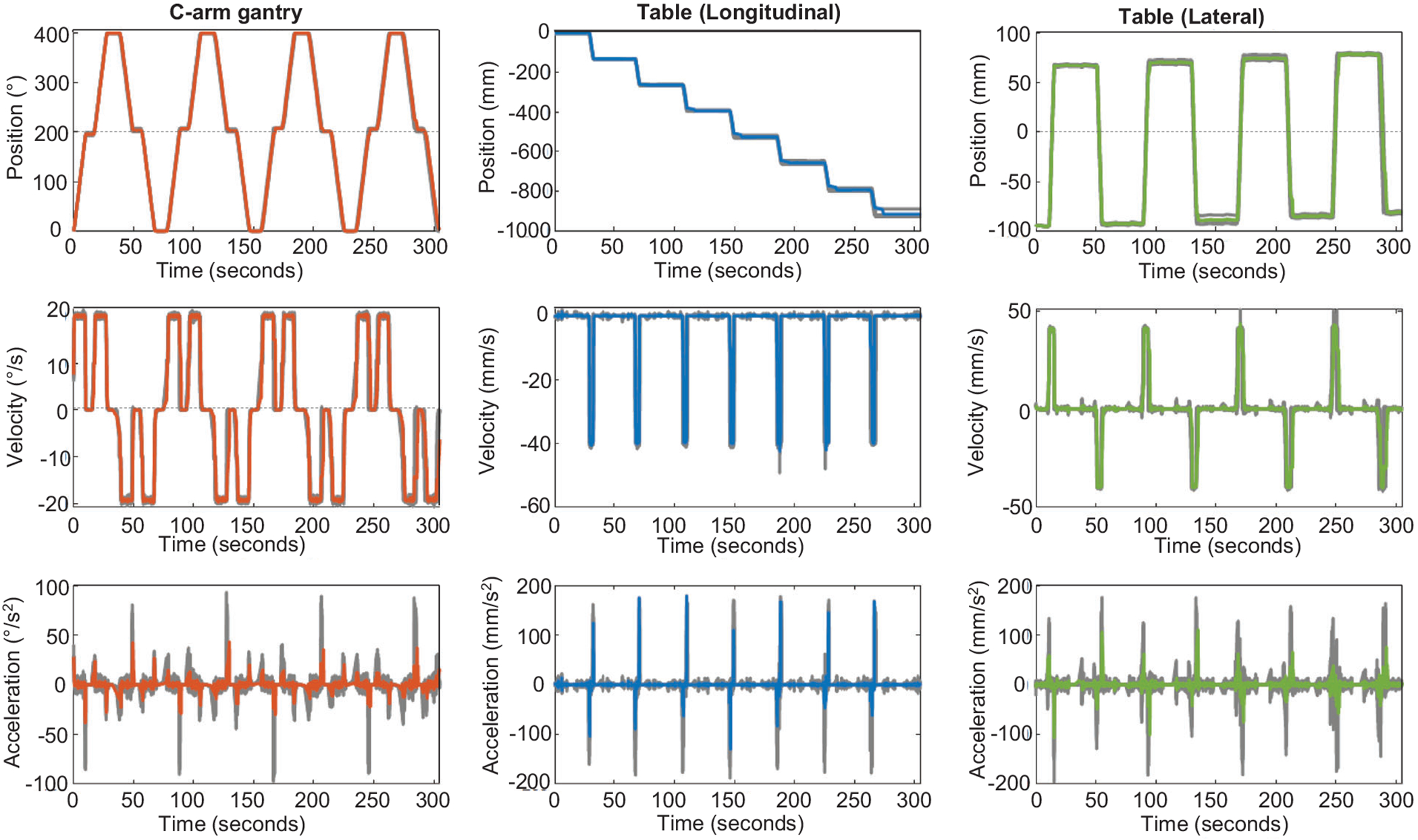
System recorded motion properties (position, velocity, and acceleration) of the C-arm and table (longitudinal and lateral) during five consecutive continuous dual-isocenter acquisitions on floor-mounted robotic CBCT system. Colored lines display the average of four scans, whereas the grey lines are the individual scans.

The position, velocity, and acceleration of the table and C-arm gantry recorded from the robotic CBCT system during four consecutive continuous dual-isocenter acquisitions are provided in Figure 3. Over the four consecutive acquisitions, the average lateral table translation was 161.38 ± 2.21 mm, the average longitudinal table translation was 130.66 ± 6.00, and the average circular arc was 202.04 ± 4.49°.

**FIGURE 4:**
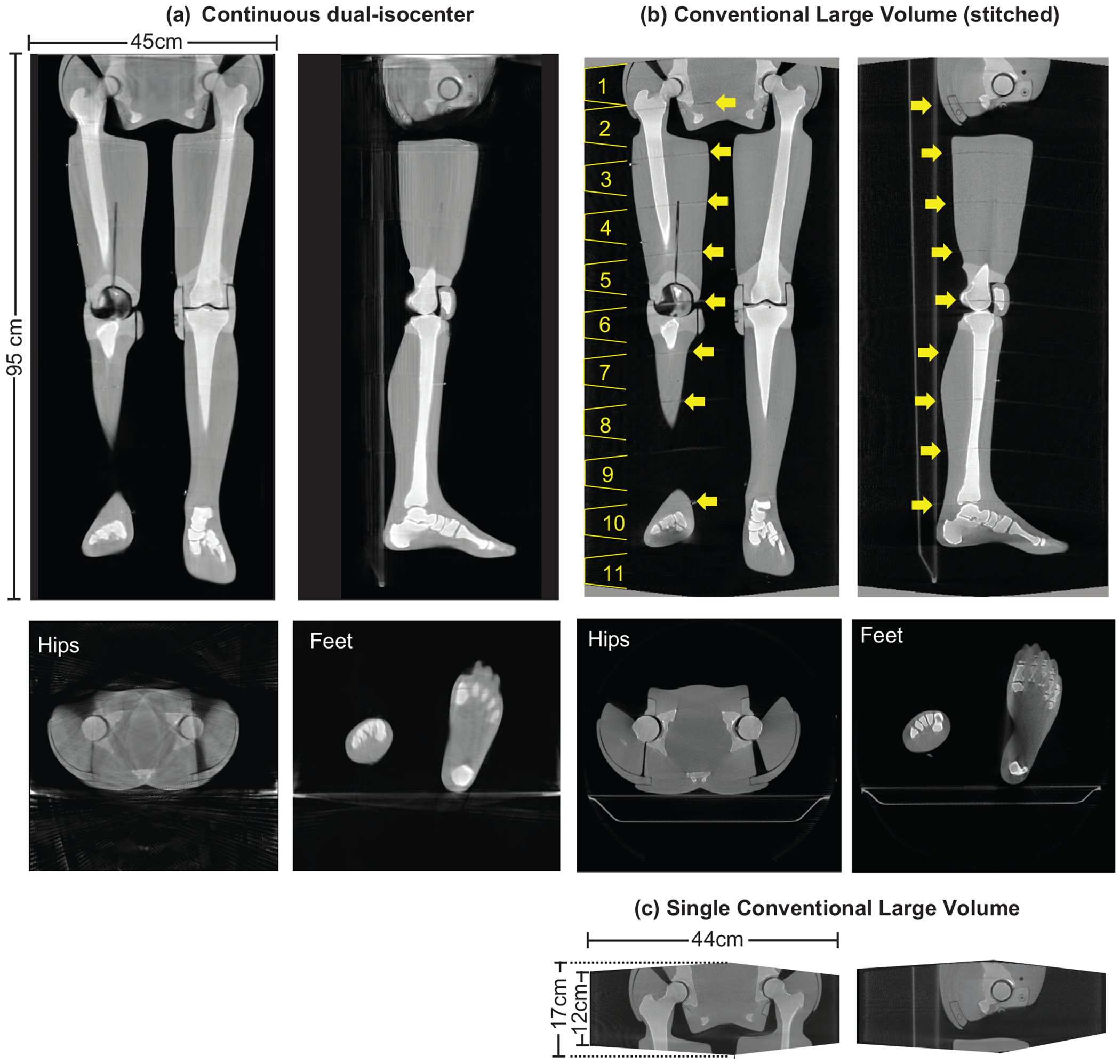
Reconstructed 3D images from (a) continuous dual-isocenter, (b) a series of stitched conventional acquisitions (numbered 1–11), yellow arrows indicate stitch points, and (c) a single conventional acquisition (coronal and sagittal slices). Grey value display: window width (W = 1756) and window level (L = −145).

Examples of the 3D reconstructed images of the whole-body phantom from hips to feet acquired with the continuous dual-isocenter and a series of conventional acquisitions stitched together are provided in Figure 4. The continuous dual-isocenter acquisition provided an imaging volume that had dimensions of length = 95 cm × width = 45 cm × height = 45 cm, reconstructed from 3066 projections. Comparatively, 11 stitched conventional acquisitions were required to match the continuous dual-isocenter imaging dimensions, reconstructed from 6006 projections.

Visually comparing the two volumes in the coronal and sagittal slices, the stitching artifacts in the conventional volume (indicated with yellow arrows in Figure 4), are no longer present in the volume reconstructed from the continuous dual-isocenter acquisition. Additionally, the bone anatomy and surrounding homogenous tissue structure appear to match in both acquisitions throughout the length of the phantom. However, examining the axial view of the hips from the continuous dual isocenter acquisition, there is an observable loss in image quality (streaking and blurring) compared to the stitched conventional volume. This is most likely due to truncation as this is not observed at the feet of the phantom, where the phantom completely resides within the imaging volume.

In terms of the quantitative image quality, the RMSD, MAPD and SSIM between the stitched conventional and continuous dual-isocenter acquisition volumes was 4.84, 6.58% and 0.99, respectively, indicating high similarity between the two volumes. Additionally, the CNR of the continuous dual-isocenter volume was 1.998 compared with 1.999 for stitched conventional volume.

## 4 | DISCUSSION

This study demonstrates the feasibility of simultaneously extending the longitudinal and lateral CBCT field-of-view using a continuous dual-isocenter acquisition implemented on a clinical robotic CBCT imaging system. The continuous dual-isocenter acquisition enables an intraoperative 3D field-of -view of L = 95 cm × W = 45 cm × H = 45 cm and demonstrated high reproducibility of the trajectory. To generate a volume with the same dimensions as the continuous dual-isocenter acquisition with conventional imaging protocols, a series of conventional acquisitions must be acquired and stitched together, requiring over double the number of X-ray projections and large increase in scan time. The image quality of the continuous dual-isocenter acquisition, measured through metrics of RMSD, MAPD, SSIM and CNR, matched that of the stitched conventional acquisitions.

Currently, the only way to implement non-circular imaging trajectories in real-time on clinical robotic CBCT imaging systems is with additional software or hardware control provided by the vendor. As such, there are a number of limitations to the experimental set up used to implement the continuous dual-isocenter imaging trajectory. These limitations have also been noted in previous novel imaging trajectory investigations using the TACS.^8,14,15^ First, there is a limit on how fast the C-arm gantry can rotate when being controlled via the TACS. Clinically, the gantry of robotic CBCT imaging systems can rotate up to 45°/s. Comparatively with the TACS, the gantry can only rotate up to a maximum of 20°/s. The velocity limit negatively impacts the scan time (currently ~5 min). If the gantry was able to rotate at velocities used clinically, the scan time could be halved to approximately 2.5 min. Further scan time reductions could be made with higher precision control of the table translation. Increasing the gantry velocity and table translation precision require increased vendor support beyond what is possible within the current experimental implementation available. Secondly, the exposure parameters between the conventional and continuous dual-isocenter acquisitions could not be exactly matched. This affects both the image quality and dose comparisons between the two scans. In terms of the image quality, the conventional acquisition protocols have been optimized to enable the highest quality volumetric image following reconstruction, including proprietary post-processing of the individual X-ray projections. Comparatively, it is not currently possible to use any of the in-built conventional volumetric protocols to perform image acquisition or reconstruction from arbitrary trajectories (i.e.,the continuous dual-isocenter). To allow acquisition from arbitrary trajectories, in-built protocols optimized for 2D fluoroscopic image viewing must be used. Prior to the start of the scan, the exposure parameters of the 2D fluoroscopic protocol used to acquire the continue dual-isocenter were set to match those of the conventional acquisition. However, the exposure parameters varied throughout the scan per the in-built automatic exposure control setting for 2D fluoroscopic acquisitions and the proprietary post-processing used by the conventional volumetric protocols could not be applied to the individual X-ray projections. These factors combine to limit the image quality achievable within the current proof-of-concept implementation and could be overcome with increased vendor support. In terms of dose comparisons, the total number of X-ray projections acquired can be used as a surrogate. Comparing the total number of X-ray projections between the stitched conventional and continuous dual-isocenter acquisitions demonstrates the potential reduction in imaging dose when implementing specialized imaging trajectories for extended field-of-view imaging. Future work could also look to optimize the X-ray projection acquisition during the continuous dual-isocenter scan, further reducing the imaging dose while maintaining image quality.

Another limitation is the complexity of the imaging target. That is, while the whole-body phantom contains an accurate representation of bone anatomy, there is no inclusion of variation in soft tissue/muscle/fat/skin, with the surrounding material of the phantom a homogenous urethane-based resin. This limits the ability to assess the full extent of the potential continuous dual-isocenter image quality. However, in previous studies that implemented the same acquisition methods and reconstruction algorithms, the reconstructed image quality from imaging animal cadavers matched that of the conventional acquisition.^8^

Finally, here we have utilized a robotic imager typically found in the operating room, however, the continuous dual-isocenter imaging trajectory could also be implemented on robotic systems designed for emergency room triage and out-patient monitoring following treatment. In emergency settings, the continuous dualisocenter imaging trajectory could be implemented to eliminate the need to transfer the patient specifically for additional volumetric imaging, allowing diagnostic imaging to occur at the first point-of -care within the hospital. In a post-treatment/out-patient setting, the continuous dual isocenter imaging trajectory could help ease the burden on equipment, allowing high-quality extended-view volumetric imaging to be completed on numerous systems. Therefore, novel imaging trajectories such as the continuous dual-isocenter have the potential to impact all stages of the treatment workflow from diagnosis to recovery.

## 5 | CONCLUSION

This is the first time a continuous dual-isocenter imaging trajectory has been implement on a clinical robotic CBCT system. As an example of potential clinical utility, the continuous dual-isocenter imaging trajectory was used to capture the entire lower extremities from the hips to feet of a whole-body phantom in a single imaging volume.

## Supplementary Material

Supporting Information - Acquisition Video

Additional supporting information can be found online in the Supporting Information section at the end of this article.
